# CADA: a teacher-facing learning analytics dashboard to foster teachers’ awareness of students’ participation and discourse patterns in online discussions

**DOI:** 10.1007/s10758-022-09598-7

**Published:** 2022-04-05

**Authors:** Rogers Kaliisa, Jan Arild Dolonen

**Affiliations:** Department of Education, University of Oslo, Oslo, Norway

**Keywords:** Teacher facing learning analytics dashboard, Asynchronous online discussions, Learning design, Participation, Discourse

## Abstract

Despite the potential of learning analytics (LA) to support teachers’ everyday practice, its adoption has not been fully embraced due to the limited involvement of teachers as co-designers of LA systems and interventions. This is the focus of the study described in this paper. Following a design-based research (DBR) approach and guided by concepts from the socio-cultural perspective and human-computer interaction (HCI), we design, test, and evaluate a teacher-facing LA dashboard, the Canvas Discussion Analytics Dashboard (CADA), in real educational settings. The goal of this dashboard is to support teachers’ roles in online environments through insights into students’ participation and discourse patterns. We evaluate CADA through 10 in-depth interviews with university teachers to examine their experiences using CADA in seven blended undergraduate and graduate courses over a one-year period. The findings suggest that engaging teachers throughout the analytics tool design process and giving them control/agency over LA tools can favour their adoption in practice. Additionally, the alignment of dashboard metrics with relevant theoretical constructs allows teachers to monitor the learning designs and make course design changes on the fly. The teachers in this study emphasise the need for LA dashboards to provide actionable insights by moving beyond *what things are* towards *how things should b*e. This study has several contributions. First, we make an artefact contribution (e.g. CADA), an LA dashboard to support teachers with insights into students’ online discussions. Second, by leveraging theory, and working with the teachers to develop and implement a dashboard in authentic teaching environments, we make an empirical, theoretical and methodological contribution to the field of learning analytics and technology enhanced learning. We synthesise these through practical design and implementation considerations for researchers, dashboard developers, and higher education institutions.

## Introduction

Improvements in technology have generated increased interest in gathering data that provide insights into how students engage with learning materials in both online and physical learning settings. By collecting, analysing, and measuring student data—a process known as learning analytics (LA)—teachers and other educational stakeholders aim to use the formative and summative feedback provided by LA to monitor, reflect on, and optimise students’ learning and the teaching process (Bodily & Verbert, 2017; Ifenthaler et al., 2018), particularly in technology-mediated learning environments, where teachers tend to struggle to monitor and support students’ active learning (van Leeuwen et al., 2019). Part of the LA research effort has been devoted to the development of dashboards, interactive visual displays that summarise and visualise information for teachers based on students’ learning patterns and interactions (Verbert et al., 2014; Few, 2013). However, the use of dashboards and the evidence of their impact on teachers’ everyday practice remain limited. This shortcoming can partially be explained by teachers’ limited involvement in the planning and design processes of LA tools (Martinez-Maldonado et al., 2020), which are typically designed by LA researchers and technology vendors, who tend to focus more on these tools’ technical aspects while neglecting their educational and pedagogical aspects (Kaliisa, et al., 2021a). Buckinghum Shum et al. (2019) and Van Harmelen and Workman (2012) argue that for LA implementation to be successful, its technical, social, and pedagogical dimensions require consideration since LA exists as part of a socio-technical system.

The goal of this study was to describe the planning, designing, implementation, and evaluation of a teacher-facing LA dashboard, herein referred to as the Canvas Discussion Analytics Dashboard (CADA). We used a participatory approach—in particular, a design-based research (DBR) methodology and principles of human–computer interaction (HCI)—to iteratively develop the dashboard with teachers based on their pedagogical needs and the LA preferences identified during the problem identification phase. The developed dashboard combines structural and content analysis to inform teachers about students’ participation, engagement, and the discourse patterns that arise from online discussions. This study makes several contributions to the technology-enhanced learning and LA literature. First, we introduce CADA, an LA dashboard that can be integrated as a plugin into Canvas and other learning management systems (LMSs) to support teachers with timely insights into students’ online discussions. Second, by evaluating CADA with seven teachers and nine courses over one year, we provide empirical, theoretical and methodological insights and lessons gained through the participatory iterative design process of CADA as a set of design and implementation principles for researchers and developers of LA dashboards.

### Background and related literature

#### Roles and challenges of teachers in online learning environments

The increasing use of digital learning tools and platforms has enabled the transformation of face-to-face courses into blended courses and courses in which all the information is delivered and accessible online (Børte et al., 2020). Learning management systems (LMSs) such as Canvas and Moodle support student learning by providing content online, allowing for online collaborative activities (e.g. asynchronous online discussions) beyond the physical classroom. As a result, teachers are expected to design learning activities and provide their students with the guidance to stimulate actions necessary for learning (van Leeuwen et al., 2019). However, teachers often struggle to monitor and support active learning among their students online (Damsa & de Lange et al., 2019). This is partially due to the large number of tasks teachers have and the amount of information produced during online learning activities, as this can overwhelm teachers, increase their cognitive load, and lessen their focus on students’ specific needs (Van Leeuwen et al., 2019). Thus, supporting tools such as dashboards could be used to inform and empower teachers with quicker insights into students’ participation and engagement patterns. This study intends to contribute to this area. In the following section, we provide a brief account of existing research on LA dashboards, paying particular attention to teacher-facing LA dashboards.

#### Supporting teachers in online learning environments through LA dashboards

LA dashboards combine automated analysis techniques with interactive visualisations for effective understanding, reasoning, and decision-making based on large, complex datasets on student activity (Schwendimann et al., 2016; Jivet et al., 2017). Teachers can use the insights gained from these dashboards as tools for evaluating and reflecting on their teaching practice (Keim et al., 2008), and track students’ social and cognitive progress (Van Leeuwen et al., 2015; Bakharia & Dawson, 2011). For example, if a dashboard provides information about the nature and context of students’ discussion topics, this information can be used to identify misconceptions and guide students in the right direction. Teacher-facing dashboards can be perceived as technological artefacts that provide indirect support to teachers during online learning activities (Rummel, 2018).

Studies on teacher-facing dashboards include Bakharia and Dawson’s (2011) work, which introduced the social network analysis pedagogical platform (SNAPP). SNAPP produces visualisations and metrics to assist with the evaluation of participation and social mode dimensions in online discussions. The Student Relationship Engagement System is another LA dashboard that allows teachers to personalise their engagement with large cohorts of students, using data from those students to inform their teaching decisions (Dollinger et al., 2019). van Leeuwen et al. (2019) developed three teacher-facing dashboards that offer different information layers (mirroring, advising, and alerting), while Herodotou et al. (2019) presented the Early Alert Indicators (EAI) dashboard, which examined the learning outcomes of more than 14,000 students in 15 undergraduate courses. Based on a sample of 559 teachers, the findings showed that most teachers who used the EAI dashboard only logged in occasionally, and their usage was inconsistent over time. This finding is consistent with Dazo et al. (2017), who examined analytic dashboard use by 14 teachers and found that most teachers accessed the analytics for only a very short time, making in-depth exploration difficult. The authors also reported that teachers struggled to interpret the data from the dashboard because the information was not presented in an actionable way, suggesting that most dashboards do not turn the patterns identified from student activities into possibilities for action (Keim et al., 2008). More recently, Martinez-Maldonado et al. (2020) followed a co-design approach to design for the effective use of translucent LA systems in the context of teamwork in a clinical simulation. The findings from four active teachers and subject coordinators showed that the proxy visualisations generated during the process helped teachers reflect on their pedagogical practices, particularly by using the visualised traces of nurses’ activity to revise the learning design. Positive outcomes were also reported by Wise and Jung (2019), who reported that teachers viewed dashboards as an important tool to support their teaching practices by informing relevant interventions and revising course design.

Despite these advances, this line of LA research still has gaps. First, while teacher-facing dashboards are becoming increasingly available, their use in teachers’ everyday practice is limited (Vieira et al., 2018). This can be partially explained by the limited involvement of teachers in the design of LA dashboards (Dollinger et al., 2019), with minimal examples of mature and transparent collaboration with stakeholders in the development of LA tools in the literature to date (Buckingham Shum et al., 2019; Wise & Jung, 2019; Holstein et al., 2018; van Leeuwen et al., 2019; Martinez-Maldonado et al., 2020). Yet, the usefulness of this technology should be measured based on its value to actual users (e.g. teachers) (Dollinger et al., 2019). Second, dashboards are only minimally aligned with learning theory (Gasevic et al., 2016), which makes it difficult to choose the nature of the data to collect and visualise to teachers (Jivet et al., 2017). This means that more work is needed to design LA dashboards grounded within the learning sciences, with the hope of increasing their relevance to teachers’ pedagogical needs. Lastly, most existing LA dashboards are stand-alone, meaning that they are not integrated within popular LMSs. This implies that teachers and researchers who are interested in using such tools must export student activity data into third-party tools, which is labour-intensive work. In real practice, given teachers’ time constraints, the use of such tools becomes impossible.

### The present study

To help close the abovementioned gaps, this paper presents a participatory DBR study that involves the co-design, implementation, and evaluation of an LA dashboard together with teachers in higher education. This study is guided by the following research questions:

RQ1: What are teachers’ experiences using CADA?

This question sought to explore the extent to which the teachers who participated in the design and implementation process of CADA found it useful for supporting their awareness of student learning in online discussions. In particular, we wanted to explore the teachers’ motivations, their reactions to and use of the dashboard features, the challenges they faced, and their suggestions for improving the dashboard.

RQ2: How can we design and implement LA dashboards that meet teachers’ pedagogical needs and expectations?

With this question, we wanted to reflect on the experiences from the different case studies to understand what works during the participatory development of LA systems with teachers, as well as the nature of design, implementation, and evaluation considerations to learn from the process and inform future research.

## The Learning analytics dashboard development process: a design-based research approach

The design of the Canvas analytics dashboard (CADA) was informed by a design based research (DBR) framework (Barab & Squire, 2004), which follows an iterative approach to exploring, designing, implementing, and evaluating innovative artefacts to solve a real educational problem based on collaboration between researchers and practitioners in authentic settings (Van den Akker et al., 2006; Reeves, 2006). DBR is often used in the learning sciences, which, using theoretical constructs as a starting point; iteratively develop the tool with stakeholders by testing it in real settings. The Learning Awareness Tools – User eXperience method (LATUX) (Martinez-Maldonado et al., 2015) also guided the iterative design stages. LATUX structures parts of the DBR process by emphasising the development of interface and awareness tools through five iterative design stages, which are briefly outlined below.

1) Stage 1: Problem identification.

The starting point for the development of CADA was to understand the challenges teachers face in their everyday practice and how LA can be used as a tool to deal with them. To meet this goal, an exploratory study with 16 teachers at two Norwegian universities was conducted. This stage was exploratory, and the results have already been reported elsewhere Kaliisa et al., 2021b). The findings from this stage showed that teachers struggled to make timely learning design changes and to understand students’ learning behaviours within online learning environments. Teachers found a need for LA that indicates student participation and discourse with online activities in a timely manner to support timely changes. These insights were used as a starting point from which to explore a range of candidate LA visualisations based on students’ online activities that could address the needs and challenges of teachers.

2) Stage 2: Creation of a low-fidelity prototype.

The next stage explored a range of candidate LA visualisations based on both checklist and process LA. These visualisations were shared with teachers as paper prototypes and grounded in theoretical concepts to address the teachers’ needs. The results from this stage are beyond the scope of this paper but have been reported elsewhere (Kaliisa, et al., 2020). In summary, the results based on the interviews conducted with four teachers showed that the paper prototypes were perceived by the teachers as informative in terms of students’ online behaviours and could provide insights into real-time course design changes. At the same time, the teachers found some of the visualisations too complex to understand and requested that they be presented in a simple and timely manner to support timely course adaptations. It is from this background that a high-fidelity prototype (CADA) that could be integrated into the same teaching environment used by teachers was developed.

3) Stage 3: Creation of a high-fidelity prototype.

**Fig. 1:**
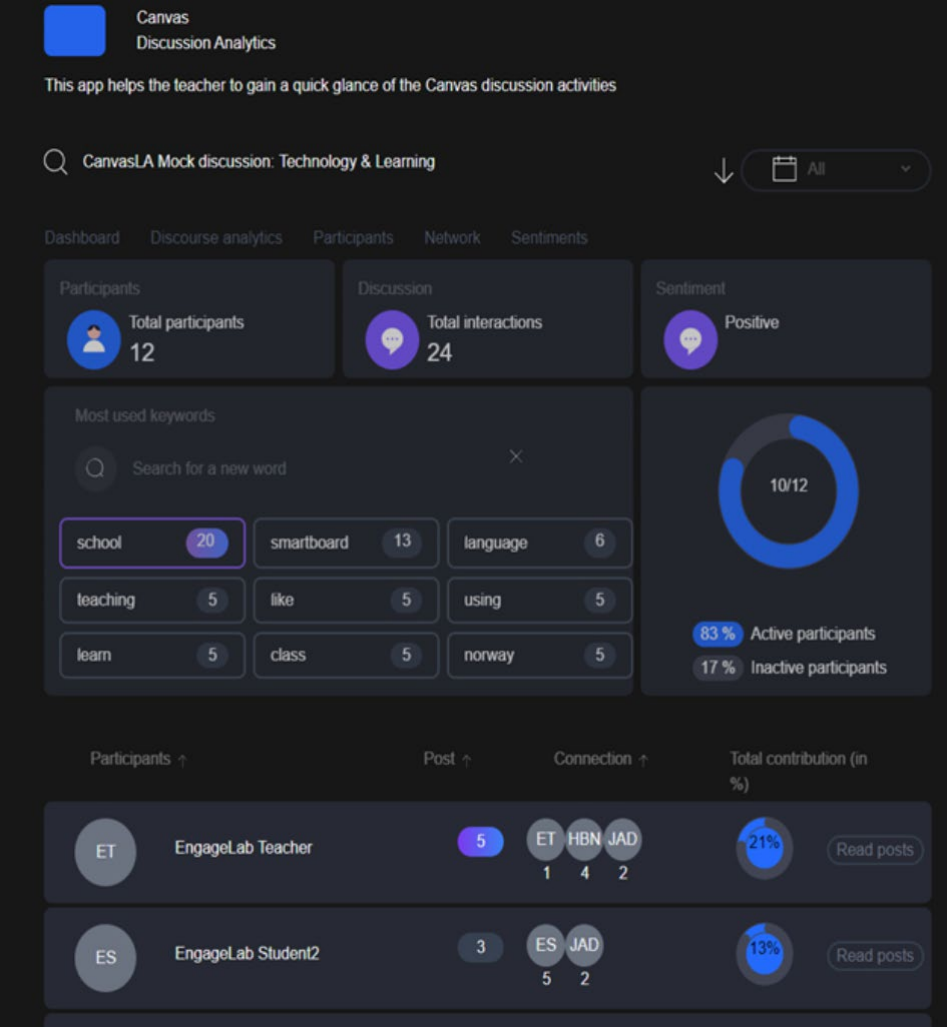
The CADA interface: General participation analytics (top), summary of the key concepts (middle), and connections between the individual students (bottom)

Based on the insights from Stages 1 and 2, an automated, high-fidelity prototype (CADA) (illustrated in Fig. 1) that sits within a Canvas course as a module or plugin was developed to automatically analyse the online discussions on Canvas in teacher-facing visualisations. To add authenticity before piloting with actual courses, we used sample data from an online discussion forum to evaluate how the users interacted with the tool. The design process was composed of a team of people with a wide range of skills and perspectives (designers, programmers, engineers, researchers, and learning scientists), as recommended by DBR (Barab, 2006). This enabled the development of a dashboard aligned with the needs of the different stakeholders while maintaining the necessary technical and design requirements. The development process went through several iterations, with changes made to the high-fidelity prototype based on the feedback gained from the teachers who participated in the initial pilot studies. In what follows, we introduce the features and the theoretical grounding of CADA, before providing details on how it was implemented and evaluated in authentic university courses.

### Introducing CADA

CADA is an LA dashboard that visualises the participation, social networks, sentiment, and concepts used by students within the Canvas LMS discussion forum on a need-to-know basis. The dashboard, which is based on the automated analysis of the discussion forum posts and interactions patterns, provides an overview for both structural and content-level analytics, which teachers can use to see students’ participation in online discussions at a glance through simple visualizations such as sociograms, which illustrate students’ discourse structures and how students share knowledge and build on each other’s contributions. Besides, the dashboard provides a detailed and fine-grained analysis of discourse, which is summarized in terms of key concepts, individual posts and sentiment. The teachers can then use the insights gained as diagnostic tool to improve their teaching and inform their learning design. The CADA interface is presented in Fig. 1, with data from one of the intervention courses.

### CADA‘s theoretical grounding

The design of CADA lent itself to principles from the learning sciences and human-computer-interaction (HCI) (Barab & Squire, 2004; Helander, 2014) to satisfy that it had a theoretical foundation and that stakeholders’ needs in real life were met (Holstein et al., 2019). Thus, the meanings, interaction opportunities, functions, and attributes associated with the dashboard were defined together with the teachers (Giacomin, 2014) but at the same time, considering theoretical perspectives. From HCI, we built on the principles of design, such as collaboration with stakeholders, signifiers, constraints, error prevention, and the reduction of cognitive load and decision-making time (Martinez-Maldonado et al., 2015). Further, CADA features elements from strategic and analytical dashboards (Few, 2006), such as the affordance for a quick overview of student engagement, thus enabling the teacher to drill into the underlying details for deeper meaning-making.

From the learning sciences perspective, the design of CADA was informed by a sociocultural perspective which is grounded in the work of the Russian psychologist Vygotsky (1978). In particular, we draw inspiration from Säljö (2002) concept of learning as the participation in, and mastery of, subject-specific discourses and practices mediated by artefacts (such as online discussions) (Säljö, 2002). Researchers in higher education have examined this issue for several decades, and active participation has been recognised as crucial to students’ learning (Børte et al., 2020). Students’ opportunities to discuss academic topics and issues together through evaluating information, reading, and commenting on fellow students’ ideas and work, as well as receiving feedback on their own ideas from fellow students and teachers, are important ingredients for constructing a deeper understanding (Black & William, 2009). Thus, teachers need to be aware of student participation in subject-specific discourses to determine the types of feedback students need to move forward in their learning trajectories (Black & William, 2009; Dolonen & Ludvigsen, 2012). In this regard, drawing on the sociocultural concepts of mediation and artefacts, CADA can be understood as a tool to communicate students’ participation and discourse patterns to the teachers and support the teachers’ cognitive efforts in understanding these patterns.

Further, the sociocultural conception of language highlights that discourse, understood both as oral and written statements, is considered an important site for understanding individual student’s learning through analyzing subject knowledge and student-student interactions (Knight & Littleton, 2015). Hence, these assumptions provided a theoretical rationale for focusing on language (as expressed in online discussions) as a key intellectual artefact, and a proxy for students’ learning that together with teachers’ pedagogical needs (identified through interviews), laid the foundation for CADA’s main learning theoretical constructs of ‘participation’ and ‘discourse.’

### Features of CADA

**Fig. 2:**
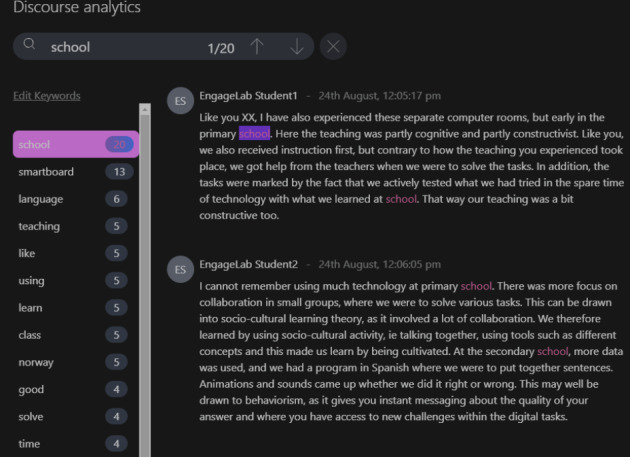
CADA’s discourse analytics showing discussion posts, key concepts and their context of use

**Fig. 3:**
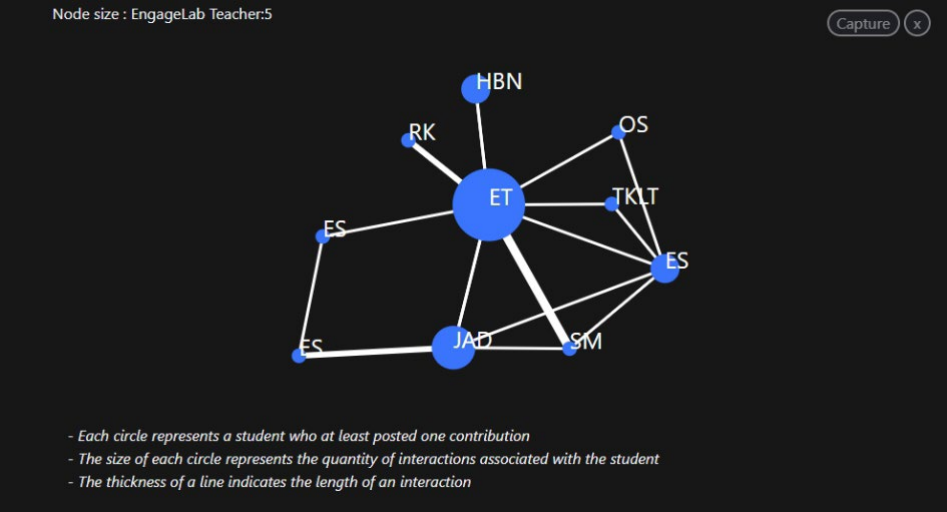
CADA’s social network diagram showing students’ interactions in a discussion forum

- *The dashboard*: This feature provides teachers with a quick overview of the discussion activity within the course and access to filtering functions, such as the percentage of active and inactive participants, the total number of interactions, and an aggregated score of sentiments for a particular thread. The information displayed through this function can be customised by the teacher according to a specific week, discussion thread, or time frame.
- *Discourse analytics*: This feature displays the key topics discussed by the students within the selected discussion forum and the context in which they were used (see Fig. 2).
- *Participation*: This function contains information on students’ participation metrics. It offers a detailed view of all the students participating on a forum, the number of posts and connections, and the percentage of total contributions for each student, which is calculated based on the size of the post. The teacher can also read all the posts associated with a particular student in one place, with time stamps on when the posts were made. Such insights could provide teachers with information such as whether and to what extent students participated in the discussion.
- *Network*: This function provides details on students’ social interactions on a discussion forum, which might be useful for teachers interested in understanding how students relate to one another at the structural level (see Fig. 3).
- *Sentiment analysis*: This function analyses the sentiments attached to each discussion post using document-level sentiment classification granularity (Kagklis et al., 2015). Here, a discussion post—the most basic unit of analysis—is categorised as expressing an overall positive, neutral, or negative opinion. Previous findings indicate that sentiment expressed in online discussions is connected to students’ performance and retention (Kagklis et al., 2015), implying that identifying students’ sentiments could help to inform the effective design of learning activities.

4) Stage 4: Implementation and evaluation of CADA in pilot and real-world classrooms.

CADA’s implementation was based on different courses and teachers’ willingness to make design changes in their courses to accommodate the rollout. Before its implementation into authentic classroom contexts, the relevant ethical procedures were completed through the national ethics committee and the university IT services team. Additionally, the researcher met with each of the teachers to introduce them to the dashboard, and instructions were provided to show them all the dashboard’s features. The first implementation phase began in fall 2020, with six teachers and five courses. The initial automated dashboard implemented into the first iteration was a simplified version that served as a starting point. This version was later updated based on feedback from the teachers, and the second iteration contained improved design and pedagogical features. For example, some teachers requested an improved interface that was easier to navigate, with clear instructions for users while they selected discussion threads. The teachers also expressed the desire to display not only the key concepts used by students, but also the contexts in which the words were used. Additionally, in our first prototype, we experimented with a variety of chart types and ways of displaying data to teachers. For example, we initially displayed the participating students in the form of a graph; however, the teachers found this overwhelming and uninformative, especially in discussions with large numbers of students (more than 50). The new changes were then built into the revised dashboard, an approach consistent with the cyclic nature of DBR (Barab & Squire, 2004). The second iteration was completed in the spring semester of 2021, with four teachers and four courses. The teachers were interviewed about their user experiences during that time. Further details about the evaluation of CADA in authentic practice are provided below.

## Study design

This study sought to gain insights into teachers’ perspectives of and experiences with the use of CADA in practice. In this regard, a qualitative approach was employed to enable the exploration of teachers’ experiences using CADA in authentic courses. Below we present the participants and the methods used to collect and analyse data about CADA’s implementation.

### Participants

The implementation of CADA involved seven teachers—four of whom had prior experience with analytics—representing nine different undergraduate- and graduate-level courses. All courses were offered online due to the coronavirus pandemic. Participation was voluntary, and only teachers teaching courses that included online discussions were involved. Three teachers were involved in both the exploratory stage (Stage 1) and the two cycles of CADA implementation; this was particularly helpful for examining how CADA had improved over time. Table 1 outlines the characteristics of the teachers who participated in the implementation and evaluation of CADA.

**Table 1:** Participants and course profiles for CADA implementation

Teacher ID	Course size and format	Level	Teaching experience (yrs)	Iteration
T1	Seminars (more than 200 students)	Bachelors	< 5	IT1
T2	Lecture & seminar (20 students)	Masters	> 5	IT1
T3	Lecture and seminar (25)	Doctoral/university staff	> 10	IT 1&2
T4	Lecture and seminar (40)	Bachelors	> 15	IT 1&2
T5	Lecture and seminar (40)	Bachelors	> 25	IT 1&2
T6	Lecture and seminar (70 students)	Bachelors	> 20	IT 1
T7	Lecture & Seminar (20 students)	Masters	< 5	IT2

### Data collection and analysis

#### Interviews

Cognitive stimulated interviews were conducted with the participating teachers, in which the interviews were held while the teachers ran through CADA’s interface. Research shows that cognitive stimulated interviews help participants to recall and reflect on the experience they are talking about, and provide researchers better insights into the way participants understand and interpret phenomenon (Dempsey, 2010; Wise & Jung, 2019). Each interview started with general questions about the teacher’s background, experience, and motivation to participate in the intervention. The main part of the interview included three sections, which were answered while the participant went through the dashboard. These sections were (1) implementation and usage, which covered questions such as how the teacher implemented CADA, how they adopted the course design, constraints during the implementation, and the effort required; (2) value-added and future usage, which included questions such as the impact of the dashboard on their teaching practice, concerns about the dashboard, suggestions for improvement, and willingness to use the tool in the future; and (3) design and implementation considerations, which covered questions such as what did and did not work, how things should be done, and lessons learnt. The development of the interview questions was guided by the study’s two research questions (e.g. what are teachers’ experiences using CADA? and, how can we design and implement LA dashboards that meet teachers’ pedagogical needs and expectations?). In addition, some of the questions were developed based on Kirkpatrick’s (2009) evaluation model, which guided the deductive analysis of the interview transcripts. The interviews, which lasted between 25 and 40 min each, were held both online (e.g. via Zoom) and physically on an agreed-upon date. The participants gave their informed consent to participate, and all the interviews were audio-recorded and then transcribed verbatim by the first author.

#### Data analysis

The analysis of the interview data was guided by an abduction approach which combines deductive and inductive elements of analysis (Linneberg & Korsgaard, 2019). First, the interviews were transcribed verbatim by the first author. They were then coded deductively according to Kirkpatrick’s (2009) model, which evaluates the results of programmes against four levels of criteria (reaction, learning, behaviour, and results). To reduce the data, we sorted the data based on Kirkpatrick’s (2009) four levels. Guided by this model, each interview response, which included a set of lines/utterances, was used as the unit of analysis. By doing so, we read the responses to establish whether they fit into the four pre-defined four levels of Kirkpatrick’s evaluation model. By following this approach, we were able to focus the analysis on those issues regarded to be important in response to the research questions. Since Kirkpatrick’s model was originally developed for evaluating training programmes, some levels were adapted using Few’s (2009) design principles for dashboards. The fully adapted evaluation criteria are provided in Appendix A. The other codes were generated inductively through a thematic analysis approach (Braun & Clarke, 2012), where the codes and later themes were developed based on the patterns from the data. While developing the themes, we focused on both the semantic and latent features of the data. This process generated five main themes (see Figs. 2 and 5) relevant to the study’s research questions. To ensure the data’s validity, the coding was performed with another researcher who was not involved in the project. Social moderation was used to settle the differences in the coding process.

## Results

### RQ1. What are teachers’ experiences using CADA?

**Fig. 4:**
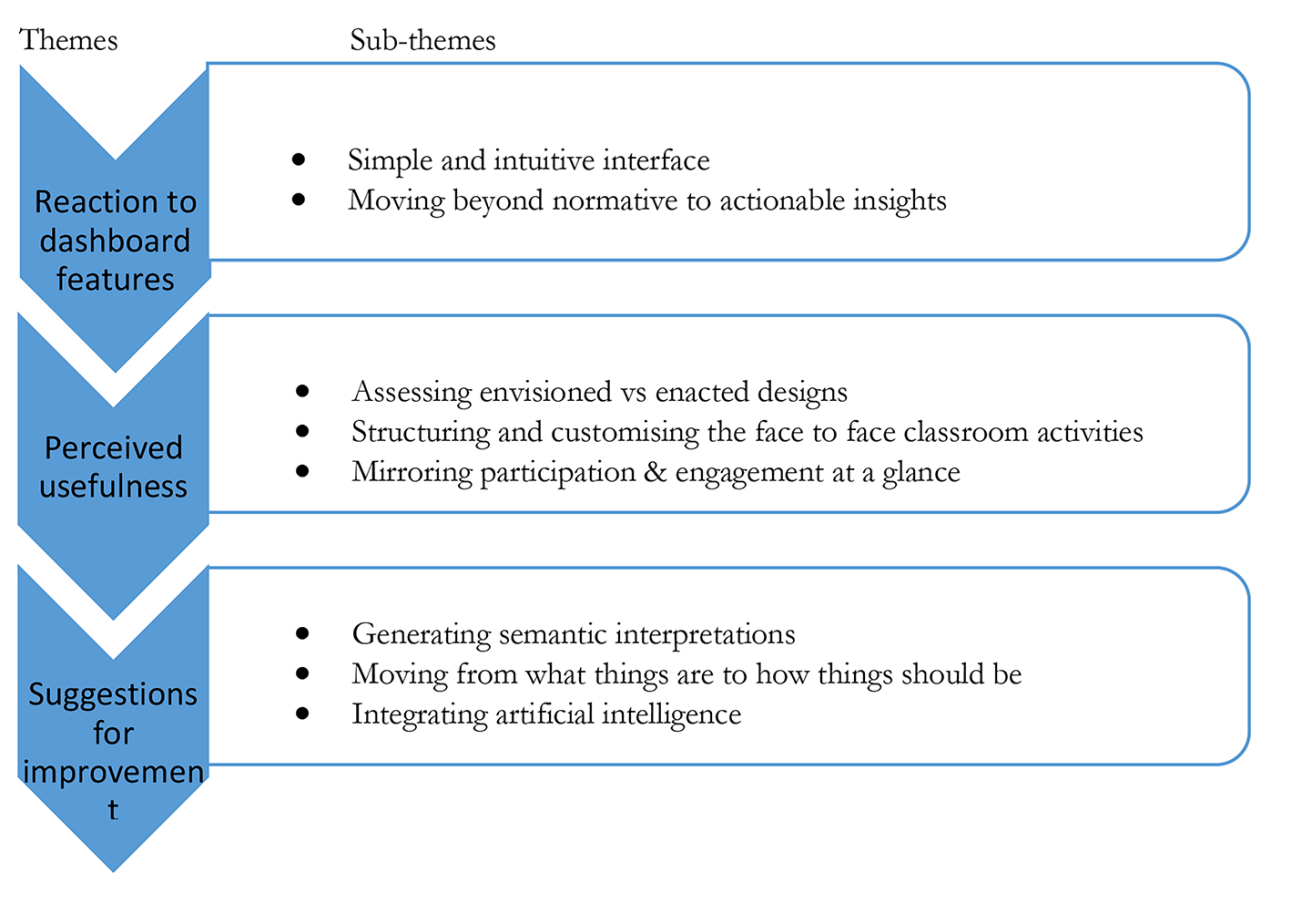
Themes that emerged from the thematic analysis of the interviews (RQ1)

Three main themes (see Fig. 4) were generated in response to the first research question.

#### Theme 1: Reaction to dashboard features

This theme answered RQ1 which sought to explore teachers’ experiences of using CADA in authentic practice. The teachers commented on the specific features of the dashboard, particularly the interface, participation, discourse, and social network features. All the teachers stated that the dashboard was very intuitive and easy to use: ‘I think this is perfect. It is very intuitive and not very fancy’ (T1IT1), and ‘It gives me what I need with no need for much data literacy’ (T3IT1). Another teacher added, ‘Honestly, the tool is very simple to use in terms of its user interface because it is just pressing a couple of buttons’ (T2IT1). Three teachers commented on how the social network analytics feature helped them see how their students were interacting. ‘The social network feature is very important, as it is related to social learning aspects’ (T5IT1). Another feature that the teachers found fascinating was the discourse analytics function (see Fig. 2), particularly the keywords section, which provided a summary of the keywords used by the students during a specific discussion. One teacher noted, ‘I think the word cloud is simple, informative, and very nice to share with the students’ (T6IT1).

Other teachers, however, questioned several of the dashboard features. For example, one teacher was not convinced about the usefulness of the discourse analytics function: ‘I would generally be more interested in phrases than individual words so that I pick out something that shows knowledge development, and this is something that you need AI or machine learning to help with’ (T5IT2). The same teacher added that while the dashboard showed how things are in terms of what is being discussed, it provides few insights on what should be done to improve students’ learning: ‘For example, if I see Student X, she is participating, but what she could do to get better is not clear. Could she use more concepts or interact more with the others?’ (T5IT2). Another commented that while the tool provides an overview of the key concepts discussed by the students, the insights do not provide a nuanced understanding of what was being discussed (T3IT1).

#### Theme 2: Perceived usefulness

*Assessing envisioned vs. enacted designs*: The teachers commented on the dashboard’s potential to provide them with information to assess the envisioned and enacted learning designs. One of them said, ‘When I saw Dysthe, which was one of the articles I had assigned for the readings, that proved to me that they had read the assigned readings and tried to integrate them into the discussion’ (T3IT2). Another stressed the importance of being able to see how the students were reacting to the intended pedagogical activities: ‘This dashboard showed me how students respond to the activities’ (T7IT2). A third highlighted that the network analytics provided information about students’ interactions with the assigned activity: ‘I can say I had to double-check the network diagram and what was in the discussion, and I realised that YES, nobody was commenting on anything, as all contributions were directed towards the original post’ (T3IT2).

*Structuring and customising face-to-face classroom activities*: The teachers stressed the importance of CADA in providing information about students’ learning processes through simple visualisations, which they leveraged to structure and customise face-to-face classroom activities. In particular, they noted the positive impact of having information such as students’ misconceptions about a topic, as this helps them to make necessary changes in their lectures and seminars. For example, one teacher noted, ‘When I looked at the discourse analytics, I realised that the students had not gone much into the key concepts. For example, they were talking a lot about “Zoom” instead of collaborating, as I expected, and later, I said, “These are things we will examine deeper later in the class” (T1IT1). According to this teacher, the insights from CADA afforded more coherence to the physical lectures. Another teacher raised similar insights: ‘The idea for me was that the insights from the dashboard helped me to structure the lesson and customise it to the things they were talking about’ (T2IT1). A third teacher added, ‘I ended up using the dashboard by summarising and synthesising the issues they had said they knew about at the beginning of the lectures, and I can say the issues visualised in the dashboard were super useful for me ahead of class’ (T7IT2).

*Mirroring participation and engagement at a glance*: A recurring theme from the interviews was the teachers’ use of CADA to indicate student engagement with a given discussion thread at a glance, as this facilitated quicker learning design decisions and saved a lot of time: ‘I sometimes looked at the tool before the lecture and skimmed through all the students’ submissions on the forum. This was quite demanding, but it was easier with the tool because I could see everything at a glance’ (T5IT2).

#### Theme 3: Future use of the dashboard and suggestions for improvement

The teachers were asked whether they were willing and ready to use the dashboard in their everyday practice, and all of them showed interest.

‘I can admit it is extra work when it comes to redesigning courses to include elements such as discussions to capture the analytics, but in the end, it is useful for the students and the teacher, so I would consider doing this again.’ (T2IT1).

‘I have used this tool for two terms now, and I can say it has been very helpful in preparing me for seminars. I will continue using it in the future.’ (T4IT2).

‘I have seen the value the dashboard provides, such as getting to know what students know ahead of the class, and I don’t think I need much pushing to use it in the future.’ (T3IT2).

The teachers also suggested improvements to facilitate future use of the dashboard. For example, one of them suggested the need to go beyond generating key concepts used in discussions to provide semantic interpretations showing the relationship between concepts. The teachers also expressed a need for CADA to extend from presenting how things are (how students interact and the concepts that they use) to include actionable insights that can inform the teacher and students how things should be. The teachers asked for predefined interventions to help them provide support to less engaged or struggling students. One of the teachers even suggested integrating artificial intelligence features into CADA as a way to improve CADA’s effectiveness.

### RQ2. How can we design and implement LA dashboards that meet teachers’ pedagogical needs and expectations?

**Fig. 5:**
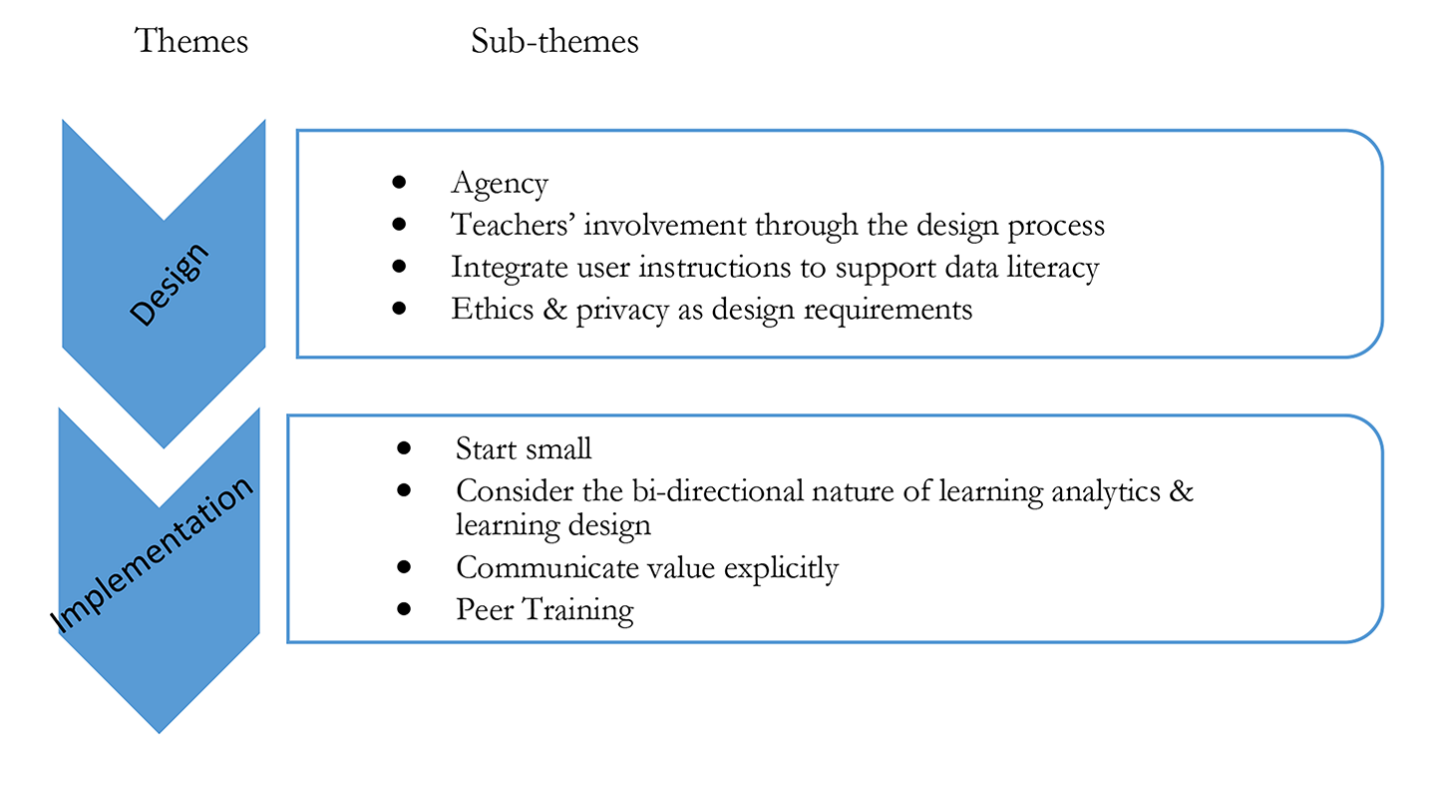
Themes that emerged from the thematic analysis of the interviews with the teachers and researcher experiences (RQ2)

To answer RQ2, we analyzed comments from the teachers and researchers’ own experiences and reflections. The analysis resulted in a set of implications that can be summarised into two main themes (Fig. 5): design and implementation.

#### Theme 1: Design considerations

This theme answered RQ2 which sought to explore how researchers can design and implement LA dashboards that meet teachers’ pedagogical needs and expectations.

*Agency*: The ‘Agency’ theme reflected how the teachers expressed the need to design LA tools with a high degree of flexibility so that they can customise them to get the kind of feedback they require. Most teachers were against the idea of designing dashboards with strict guidelines. ‘I think using strict guidelines does not work because teachers, including myself, all have different ways of and plans for teaching’ (T2IT1). Another teacher emphasised the value of agency while designing LA dashboards for teachers: ‘I think it is important that, as a researcher, you do not state what should be done with the tool but instead offer options from which the teachers can choose, which is a way of giving them agency’ (T3IT2). Another added, ‘If there is a way to allow the teacher to edit the ideal state where you would like your students to be at the end of the course, and the tool helps to illustrate this process, that could be very important’ (T5IT2).

*Teachers’ involvement throughout the design process*: A critical problem with many LA systems is that their design follows a top-down process in which researchers and developers make decisions without involving the intended users (Dollinger & Lodge, 2018). Our design of CADA was motivated by the challenges the teachers mentioned during the problem identification stage, as well as the feedback received during the different iterations in which the teachers provided feedback on the early prototype. Consequently, the teachers expressed satisfaction with their engagement in the design of the dashboard: ‘I remember you interviewed me two times for the first version, and I was surprised to see this much-improved version of the interface with some of the feedback I gave put into consideration in the new designs’ (T5IT2).

##### Integrate user instructions to support data literacy

While the teachers were generally happy about the dashboard’s interface, they expressed the need for clear instructions to guide them in using the dashboard. ‘I think having video tutorials and screenshots to guide teachers could be helpful’ (T5IT2). This points to the issue of data literacy, which has been discussed in the LA literature (Kaliisa et al., 2021a). In other words, to favour the wide adoption of LA tools, particularly among non-data experts such as teachers, the tools need to be designed with user-friendly instructions, as teachers may lack data literacy skills and contextualised affordances on how to take advantage of these data.

##### Ethics and privacy as design requirements

To integrate CADA into the intervention courses, we sought clearance from the university’s legal team and study administration because in order to implement new plugins into Canvas at our university there is a need to adhere to various privacy and ethical requirements for managing personal information such as a legal basis, secure data storage and correct access privileges. Besides, in light of the increasing datafication of and surveillance in education, as well as general concerns about privacy and ethics, we found no resistance from the teachers in using the dashboard once they were assured that the relevant privacy and ethical considerations had been met.

#### Theme 2: Implementation considerations

##### Start small

One of the key implementation considerations highlighted by the teachers was the need to conduct case studies with fewer teachers before scaling up the intervention. During the initial pilot stage, as researchers, we started working with only two teachers, who later acted as disseminators and ambassadors by suggesting the dashboard to other teachers. One teacher commended the approach we used in this study, saying, ‘It is very smart to start with a few teachers before moving to others to gain some momentum… the few teachers will let others know how the tool works, and this is what you have done already’ (T5IT2).

##### Consider the bi-directional nature of learning analytics and learning design

The teachers pointed to the need to consider the connection between LA tools and their learning design practice. Some argued that researchers, as they intend to introduce LA tools such as dashboards, need to plan and work with teachers to ensure that the tools align with the intended course design practices. One teacher commented, ‘It is important for teachers to be aware of how the tool plays into their own design process ahead of time. This makes it easier for the teachers to embed the tool into their own teaching’ (T3IT2). This was demonstrated in the case studies that were involved in the implementation of CADA; the teachers redesigned some elements of the course to gain insights into specific parts of it and to gather relevant LA. When asked about the future use of the tool, one teacher answered, ‘I think I would like to use the tool again, but I will have to make a few changes to the course to allow more posts and better analytics’ (T6IT1).

##### Communicate value explicitly

One of the overarching themes across the interviews was the need for explicit communication regarding the value of the proposed LA tools, particularly for improving the teachers’ teaching practices. The teachers argued that once the value of the tool is well communicated, its adoption into practice becomes easier. ‘I understand getting the teachers to commit is an issue, but once you find the teachers who are envisioning a new thing and they understand the value of the analytics tool, then it is easy’ (T3IT2). Since research has emphasised that the usefulness of the technology should be measured according to its value to actual users (Dollinger et al., 2019), it is critical for LA researchers to communicate the value of dashboards explicitly.

##### Peer training

When asked about the conditions necessary to support the use of CADA in future practice, the teachers pointed out training as a key requirement. In particular, the teachers suggested that the training be done in groups to enable peer support during the implementation. ‘The reason why I say it should be in groups is that teachers always learn from each other and ask questions about pros and cons. It is also the interaction and how people think together that could generate useful ideas for further development of LA tools’ (T3IT2).

## Discussion of the findings

The aim of this study was to follow a participatory approach to co-design, implement, and evaluate an LA dashboard with teachers to help gain insights into students’ participation and discourse within online discussions. To achieve this aim, we established two research questions: (1) What are teachers’ experiences using CADA and (2) How can we design and implement LA dashboards that meet teachers’ pedagogical needs and expectations?

Regarding RQ1 on teachers’ experiences of using CADA, the teachers who participated throughout the design process were positive about the dashboard features, and they showed interest in using it in their future practice. In particular, they stressed the importance of CADA in providing information about students’ learning processes through simple visualisations, which they leveraged to gain a more nuanced understanding of how particular terms were used by the students and, where necessary, how the identified misconceptions were used as a basis on which to structure and customise face-to-face classroom activities. Previous research has reported that very few LA systems, present social learning analytics visualisations (e.g., social networks, discourse analytics) in real-time to support teachers’ learning design decisions (Kaliisa et al., 2022). By enabling teachers to make small changes to their physical classroom lectures based on automated social network and discourse analytics visualisations, CADA showed the potential of LA dashboards to improve teachers’ learning design practices without solely relying on summative assessments and end-of-semester evaluations, which the literature has reported as untimely and relatively biased (Bennet et al., 2015). Furthermore, the literature on teachers’ roles in online learning environments has reported capturing students’ participation and discourse as a particularly difficult task for teachers (van Leeuwen et al., 2019; Børte et al., 2020). With CADA, the teachers appreciated gaining insights into students’ participation ahead of the physical classes. An important implication for researchers is that well-designed dashboards, aligned with teachers’ pedagogical needs, and providing timely and automated visualisations, have the potential to support teachers in their challenging instructional roles, particularly in technology-supported online learning environments (Wise & Jung, 2019).

Although the teachers’ overall impressions of CADA were positive, some questioned the usefulness of several of its features. For example, one teacher was not convinced about the usefulness of the discourse analytics function, questioning the value of displaying the key concepts discussed by the students, which did not provide a nuanced understanding of what they were discussing. Others felt that the analytics presented by CADA only showed how things are in terms of what is being discussed, with fewer insights into what should be done to improve students’ learning. Meanwhile, CADA’s main purpose is to support teachers’ awareness of students’ participation and discourse at a glance, and teachers use this as a baseline to make pedagogical decisions. However, we also recognise that increased awareness may not be enough for teachers to intervene since information from dashboards is usually presented in a minimally actionable way (Dazo et al., 2017). With this in mind, if we are to support teachers in fulfilling their ethical obligation to act (Prinsloo & Slade, 2017) based on information from dashboards, it is critical that teacher dashboards go beyond the normative to include actionable insights to support teachers’ decision-making processes. In other words, to increase the relevance of dashboard analytics, they should be able to provide some hints (Kasepalu et al., 2021) regarding what teachers need to do. This reflection stresses the increasing need to align LA and artificial intelligence (Kasepalu et al., 2021). Holstein et al.’s (2019) work emphasises the power of dashboards by moving beyond descriptive analytics and mirroring dashboards to those that provide teachers and students with timely feedback and recommendations (Camacho et al., 2020).

In relation to RQ2, the findings highlight several design and implementation considerations for LA researchers and technology developers. First, agency and control were identified as key to supporting the adoption of LA by teachers. As reflected under the ‘Agency’ theme, the teachers expressed the need to be able to configure and choose which indicators and information they need from the system. As stated in earlier studies (Roberts et al., 2017; Shibani et al., 2020), these findings indicate that teachers need control over what LA systems provide, and this can only be achieved by engaging them actively in the design process. Moreover, given the different institutional and disciplinary contexts under which teachers work, designing customisable and adaptable LA systems cannot be overemphasised. In this regard, we plan to add features to CADA that allow teachers to choose the nature of the indicators on which they wish to focus.

In addition, responding to RQ2, the findings showed the value of involving teachers in defining their pedagogical problems and later suggesting LA solutions to deal with the problems. A key challenge identified in LA studies is the design of LA systems that are technically sound but pedagogically weak (Kaliisa et al., 2021a). In this study, we started by identifying teachers’ pedagogical challenges (e.g. difficulties in monitoring participation and discourse patterns) and later suggested indicators that could capture participation and discourse together with teachers. We proceeded by identifying relevant LA analytical techniques (e.g. social network and discourse analysis) to analyse the online discussion forums in a way that made sense to the teachers, iteratively evaluating the relevance of the analytics from these techniques with the teachers before they were implemented as features in the CADA dashboard. The teachers reported feeling motivated to use the tool, which they perceived as a product of the participatory process rather than an imposition. Besides, CADA is an example of a practice-oriented system intended to directly impact teachers’ everyday practice since it is born within the immediate context of use and co-designed with teachers who are the intended users. In this way, unlike LA systems developed based on experimental studies, CADA and the approach taken in this paper highlights an effort to limit the gap between LA research and practice, and to increase the ownership and relevance of the analytics tools presented to the teachers.

Meanwhile, the process of involving teachers and other stakeholders in the design and implementation of LA systems is without challenges. For example, while some of the participating teachers had some knowledge of LA or other educational technologies (n = 4), others had no experience (n = 3). Thus, teachers with limited working knowledge of LA struggled to make sense of some of the analytics and provided limited feedback regarding how CADA could be improved. A design challenge posed to LA researchers and designers is to determine how to find negotiated points (Dollinger et al., 2019) when working with multiple stakeholders with varying levels of expertise. Additionally, even though teachers who were well-versed in LA systems and other educational technologies provided suggestions on how to improve CADA, not all the demands were implemented, as some were found to be less technologically and ethically feasible, and beyond the researchers’ goals and resources. Again, this finding points towards the dilemma of balancing the needs of different stakeholders in participatory research during the design and implementation of LA tools.

Lastly, the current study showed that it is critical for researchers to consider the introduction of LA tools as new technologies for teachers by providing appropriate support in form of training and exemplars. It is also important that researchers allow enough time for the teachers to learn and decide how to integrate the tools in their everyday practice since LA tools usually come with underlying epistemological assumptions (Knight et al., 2014), which might not align with teachers’ own pedagogical needs. In practice, providing the necessary support to teachers might not be simple due to logistical challenges. Thus, we recommend that researchers and institutional leaders interested in LA adoption, start with small initiatives by involving a few teachers who could in turn become the champions and support local communities of practice by spreading the word about an existing tool to other teachers, and subsequently, move towards institutional adoption (Heredotou et al., 2019; Tsai et al., 2018). In the following section, we outline the key recommendations arising from the findings that should be taken into account by researchers, LA developers and higher educational institutional managers.

### Practical recommendations for researchers, LA developers and institutions

- LA dashboard designers and researchers should prioritise giving teachers control while designing LA systems that allow for insights into tool design for local actionability (Wise & Jung, 2019; Buckingham Shum et al., 2019). In particular, it is critical that the design of LA systems align with teachers’ conceptualisations of their courses (e.g. presenting analytics based on the course modules), an aspect that underscores the bi-directional nature of LA and course designs.
- Researchers and dashboard designers should integrate automated feedback systems that support the actionability of the insights gained from the dashboards. If this is not done, teachers’ cognitive loads might increase while they are trying to interpret the analytics from the dashboard, which might discourage uptake in their everyday teaching practice.
- Researchers should be explicit about their own perspectives and goals, and transparent about certain implementation constraints to avoid challenges resulting from ignoring some of the ideas provided by stakeholders (e.g. teachers, students). One way to achieve this is to maintain close communication and dialogue between different stakeholders throughout the design process.
- In light of the increasing datafication of and surveillance in education, as well as general concerns about ethics and privacy (Howell et al., 2018), we moot the need for LA researchers and designers to not only emphasise the technical aspects of dashboards but also to consider the issues of privacy and ethics while defining the protocols for dashboard designs. To properly ensure the protection of personal information there is a need to adhere to a proper legal basis, secure data storage and proper access privileges to data and visualisations. For example, interfaces can be designed with a possibility to change views, where teachers can hide student identity before sharing LA visualisations in the classroom.
- Researchers and higher education institutions should embrace the fact that developing LA systems is a team effort. CADA was developed with input from researchers, teachers, students, engineers, programmers, study administrators, legal teams and ethical committees. This highlights the need for well-coordinated efforts involving several stakeholders prior to the design of LA systems.

### Limitations and opportunities for future research

One of the limitations of this study was the class sizes used during the evaluation of the dashboard. Most courses had between 20 and 40 students, which means that the teachers did, not easily recognise the actual benefits of CADA, particularly in large courses. Second, while the study included seven different courses and teachers—slightly more than most existing LA studies—this number was not large enough to allow for the generalisation of the user experiences captured. Most teachers wanted assurance on the potential of the dashboard before making design changes in their courses to accommodate the dashboard, which affected the number of teachers at the pilot stage. Third, during CADA’s implementation, dashboard updates were ongoing, and features such as sentiment analysis were fully integrated towards the end of the pilot studies. This means that some teachers experienced different functionalities at different points of use. Lastly, the evaluation of CADA was based mainly on user interviews and researcher observations of courses in which this was possible. While user experiences can provide lived experiences, it can be difficult to understand the actual impact and usage of the tool. Thus, we seek to expand this work by engaging with teachers instructing more popular courses and using more fine-grained methods to analyse the actual use and impact of dashboards. Researchers such as Herodotou et al. (2021), for instance, have employed finer-grained methods of data collection, such as eye-tracking, log analysis, and screen capture videos, to gain additional insights into teachers using dashboards in practice. Despite these limitations, this study provides insights into a participatory process among teachers to develop an LA dashboard, as well as relevant design, implementation, and design considerations that other researchers could leverage while developing LA tools. Going forward, we plan to share developer codes to expand the use of CADA across contexts. We also plan to continue developing CADA features based on the feedback from the pilot studies before CADA is released as a plugin in Canvas and other LMSs; this will allow other researchers, teachers, and institutions to use CADA for their own work.

## Conclusions

This study’s contribution is fourfold. First, from a *practical* point of view, the study offers an artefact contribution in the form of CADA. Teachers have already started using CADA in their everyday teaching practice; thus, CADA is contributing to demonstrable changes among practitioners (Barab & Squire, 2004). CADA is unique in that it can be directly integrated into several LMSs as a plugin. Thus, it represents a step forward in fulfilling the goal of LA to support and inform timely learning design decisions. Second, the study has a *theoretical* contribution by demonstrating how LA researchers and designers can utilise theoretical constructs (sociocultural perspective) to design theoretically sound systems that align with teachers’ pedagogical needs. Third, the study has an *empirical* contribution by generating empirically grounded design and implementation considerations that can be utilised by researchers, technology developers and higher education institutions interested in the research, design and adoption of LA dashboards. *Methodologically*, this study has provided a demonstrable and successful process of using participatory approaches (e.g. design-based research and human-centred learning analytics) over two iterations with key stakeholders to improve the value and uptake of LA systems. While we do not advocate for the use of the CADA dashboard developed in this study, we hope that the insights from teachers’ use of CADA in authentic practice, as well as the practical design and implementation considerations derived from this series of case studies, could be useful platforms upon which other researchers planning to develop and implement LA tools can build.

## Data Availability

Not applicable.
